# Effect of Anthropogenic Landscape Features on Population Genetic Differentiation of Przewalski's Gazelle: Main Role of Human Settlement

**DOI:** 10.1371/journal.pone.0020144

**Published:** 2011-05-20

**Authors:** Ji Yang, Zhigang Jiang, Yan Zeng, Mardan Turghan, Hongxia Fang, Chunwang Li

**Affiliations:** 1 Key Laboratory of Animal Ecology and Conservation Biology, Institute of Zoology, Chinese Academy of Sciences, Beijing, China; 2 Graduate School of the Chinese Academy of Sciences, Beijing, China; Natural History Museum of Denmark, Denmark

## Abstract

Anthropogenic landscapes influence evolutionary processes such as population genetic differentiation, however, not every type of landscape features exert the same effect on a species, hence it is necessary to estimate their relative effect for species management and conservation. Przewalski's gazelle (*Procapra przewalskii*), which inhabits a human-altered area on Qinghai-Tibet Plateau, is one of the most endangered antelope species in the world. Here, we report a landscape genetic study on Przewalski's gazelle. We used skin and fecal samples of 169 wild gazelles collected from nine populations and thirteen microsatellite markers to assess the genetic effect of anthropogenic landscape features on this species. For comparison, the genetic effect of geographical distance and topography were also evaluated. We found significant genetic differentiation, six genetic groups and restricted dispersal pattern in Przewalski's gazelle. Topography, human settlement and road appear to be responsible for observed genetic differentiation as they were significantly correlated with both genetic distance measures [*F_ST_*/(1−*F_ST_*) and *F′_ST_*/(1−*F′_ST_*)] in Mantel tests. IBD (isolation by distance) was also inferred as a significant factor in Mantel tests when genetic distance was measured as *F_ST_*/(1−*F_ST_*). However, using partial Mantel tests, AIC*_c_* calculations, causal modeling and AMOVA analysis, we found that human settlement was the main factor shaping current genetic differentiation among those tested. Altogether, our results reveal the relative influence of geographical distance, topography and three anthropogenic landscape-type on population genetic differentiation of Przewalski's gazelle and provide useful information for conservation measures on this endangered species.

## Introduction

The potential effect of landscape features on dispersal and population differentiation of wild animals has been well recognized [1]. With the rapid expansion of human population and associated land use, concerns have been raised about the influence of anthropogenic landscape features because they could impede gene flow, lead to population isolation and genetic differentiation [2], [3], and reduce genetic variation and evolutionary potential [4], [5]. This research field is now a focus in population and conservation genetics [6], and many recent studies have made much progress including in invertebrates [e.g. 7], amphibians [e.g. 8], reptiles [e.g. 9] and mammals [e.g. 10]. However, despite some studies on the genetic effect of certain anthropogenic landscape type such as road [5], [11], most previous researches in this field have tended to present the total effect of different anthropogenic landscapes. As not every type of landscape features exert the same effect on a species, it is necessary to assess their relative effect for species management and conservation. Moreover, given that landscape effects are a function of both environmental features [12], [13] and the biological characters of a species [14], [15], studies across more diverse taxa and regions are needed to fully understand the influences of man-made landscape features on wild animals. In central Asia, many ungulates are threatened or endangered [16], yet little is known about how anthropogenic landscape features affect these animals. Here, we carried out a landscape genetic study on Przewalski's gazelle (*Procapra przewalskii*), which inhabits a human-altered area on the Qinghai-Tibet Plateau in central Asia, to investigate the relative genetic effect of different types of landscape features.

**Figure 1:**
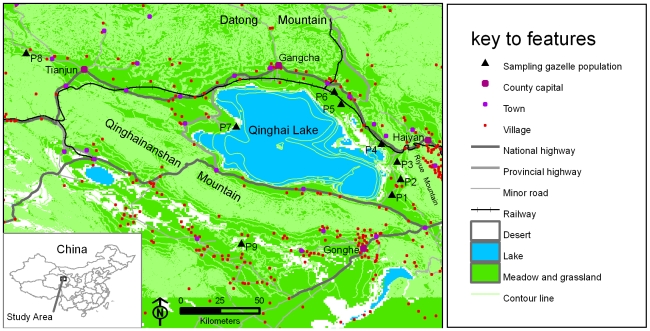
Map of nine sampling populations and landscape features. There are three main types of anthropogenic landscape features (human settlements including county capitals, towns and villages; roads including national highways, provincial highways and minor roads; railways) in the study area.

The Przewalski's gazelle is one of the most endangered antelope species in the world [17]. It is endemic to China and a flagship species in the eastern part of the Qinghai-Tibet plateau [18]. The species once inhabited Gansu, Inner Mongolia, Ningxia and Qinghai Provinces, China but has experienced a sharp population reduction [19], [20]. Now, several hundred of individuals survive in several localities in Qinghai Province near Qinghai Lake (Figure 1) [21], [22]. Accordingly, it is listed as ‘Endangered’ under the IUCN Red List (2008) [23] and as a Category I National Key Protected Wild Animal Species under the Wild Animal Protection Law of China [20]. Przewalski's gazelle inhabits on a vast grassland without obvious natural barriers between most populations, and the gazelle has high locomotive ability (45–55 kph) [24] and relatively short inter-population distances (8.5–205.8 km, Table 1), however, the total home range sizes of the gazelle are about 800 hm^2^ under human disturbance [25], and previous study based on mitochondrial DNA (mtDNA) control region sequences revealed unexpected significant genetic differentiation among four populations [26]. The authors inferred that anthropogenic barriers probably influenced genetic differentiation, but there was no explicit analysis to support the inference [26]. Since new molecular markers and analytic methods are now available, we ask the following questions: what level of genetic differentiation is there in Przewalski's gazelle on the basis of microsatellite markers? Which factors caused genetic differentiation, anthropogenic or other factors? How important is the relative effect of each factor? Here, we considered five factors which possibly influenced genetic differentiation, including human settlement, road, railway, geographical distance and topography (lake and mountain). Given that the distribution and habitat of the gazelle are fragmented by many man-made landscape features (Figure 1), we hypothesize that anthropogenic landscape features influenced population genetic differentiation of Przewalski's gazelle. We expected that significant genetic differentiation would be observed, and certain types of anthropogenic landscape features would significantly correlate with the genetic distances.

**Table 1:** Matrix of geographical distances (upper diagonal) and matrix of categorical distances of topography (lower diagonal) between nine populations.

Population	P1	P2	P3	P4	P5	P6	P7	P8	P9
P1	—	11.49	23.19	33.28	63.53	72.00	91.06	205.81	83.13
P2	0	—	12.68	23.82	55.51	63.89	89.51	204.32	90.82
P3	0	0	—	11.47	43.55	51.81	82.51	196.66	94.27
P4	0	0	0	—	32.10	40.34	74.25	187.41	95.08
P5	0	0	0	0	—	8.47	55.23	161.53	102.16
P6	0	0	0	0	0	—	54.57	156.75	107.42
P7	1	1	1	1	1	1	—	114.89	74.70
P8	1	1	1	1	1	1	0	—	162.61
P9	1	1	1	1	1	1	1	1	—

Geographical distances were represented as Linear Euclidean distances in kilometres. Topography included lake and mountain, categorical distances described the presence (1) or absence (0) of the topographic feature (lake or mountain) between two populations.

In this study, we used 13 nuclear microsatellite loci and landscape genetic methods to test our hypothesis. The aims of this study are: (1) to estimate population genetic differentiation and dispersal pattern of the gazelle; (2) to identify the factors responsible for observed genetic differentiation and to reveal their relative effect. The results provide useful information for conservation of Przewalski's gazelle and, more broadly, yield empirical data for understanding the genetic effect of anthropogenic landscape features on wild ungulates across Eurasia grasslands.

## Methods

### Ethics Statement

Our research on Przewalski's gazelle in the Gonghe Basin and Qinghai Lake Basin was approved by the Chinese Wildlife Management Authority and conducted under the Wildlife Protection Law of the People's Republic of China.

### Study area and sample collection

This study was conducted around the Qinghai Lake (98°26′–100°54′E, 36°13′–37°36′N). Qinghai Lake is the largest inland saline lake in China, and a perennial lake that freezes in winter. The lake has an area of 4300 km^2^ and an average water depth of 16 m (max. 28 m). The study area is 2900–3800 m above sea level, surrounded by several mountains. There are three main types of man-made landscapes fragmenting the distribution and habitat of the gazelle: human settlements (county capital, town and village), roads (national highway, provincial highway and minor road) and railways (Figure 1). Their characteristics and age were obtained from local chronicles of the four counties in the study area [27]–[30], literatures [31]–[34], the Fifth National Census Data (2000) and field survey (Table 2). Samples of Przewalski's gazelle were collected from all known populations (P1–P9, Figure 1), including Yuanzhe (P1, about 40 individuals), Hudong (P2, about 100 individuals), Ketu (P3, about 60 individuals), Shadao (P4, about 10–20 individuals), Ganzihe (P5, about 20–30 individuals), HerG (P6, about 80 individuals), Bird Island (P7, about 10–20 individuals), Shengge (P8, about 80 individuals), and Qiejitan (P9, about 70 individuals) [21], [22]. Population P9 is located in the Gonghe Basin and probably isolated from other populations by Qinghainanshan Mountain. The remaining eight populations are located in the Qinghai Lake Basin surrounded by lofty mountains, with Datong Mountain on its north, Riyue Mountain on its east and Qinghainanshan Mountain on its south (Figure 1). Skin samples were collected from wolf-killed gazelles discovered in the field from 2004–2007 and stored dry at −20°C. Fresh fecal samples were collected in winter (Nov–Dec 2006) to reduce genotyping error rates [35] and were preserved in 100% ethanol. Given our research experience with this species, we have detailed knowledge of the locations known to support the gazelle populations. After a herd of gazelles was located and observed for some time, we approached the gazelles and collected all fresh feces from the ground. Because P4 and P7 have very small population sizes, collecting fresh fecal sample is especially difficult for the two populations. Locations for each sample were recorded using a handheld global positioning system (Garmin Etrex Vista C, Garmin Ltd.).

**Table 2:** The characteristics and age of anthropogenic landscape features in the study area.

Anthropogenic landscape feature		Characteristics	Age
Human settlement	county capital	about 5 km^2^ and 8000 persons	at least 1000 years
	town	about 1 km^2^ and 1000 persons	
	village	about 0.2 km^2^ and 200 persons	
		totally 65 km^2^ and 100665 persons	
Road	national highway	about 8 m width and 60 vehicles per hour by day, 38 bridges, 592 underpasses, no fence	within 71 years
	provincial highway	about 6 m width and 30 vehicles per hour by day, 28 bridges, 214 underpasses, no fence	within 60 years
	minor road	about 4 m width and 10 vehicles per hour by day, 10 bridges, 40 underpasses, no fence	within 51 years
Railway		about 3 m width and 3 trains per hour, 31 bridges, 132 underpasses, partly fenced	within 50 years

Human settlement characteristics were obtained from local chronicles [27]–[30], literatures [31]–[34], the Fifth National Census Data (2000) and field survey. Road and railway characteristics were acquired from local chronicles [27]–[30], local railway station record and field survey. The age of anthropogenic landscape features were obtained from local chronicles [27]–[30] and local people.

### DNA extraction and microsatellite amplification

Genomic DNA from skin samples was isolated using standard proteinase K digestion and phenol/chloroform extraction procedures [36], followed by a UNIQ-10 column (Sangon) purification. Fecal samples were extracted using QIAamp DNA Stool Mini Kit (QIAGEN) according to manufacturer protocol. Extraction blanks were used as negative controls. We used 13 species-transferred microsatellite markers (AF5, AGLA226, BM1225, CSSM43, IDVGA39, JAB8, RBP3, RT1, T156, TEXAN15, TGLA10, TGLA122 and TGLA378) in this study [37], [38] on the basis of consistently amplifying clear and polymorphic products in fecal samples. One homozygote of each locus was sequenced to confirm it was a short tandem repeat (STR) in Przewalski's gazelle. Then, forward primers of the 13 markers were fluorescently labeled with FAM, HEX and TAMRA. We used a reaction volume of 10 µl, containing approximately 10 ng of genomic DNA, 0.2 µM of each primer, 0.1 mg/ml of bovine serum albumin (BSA, Biolabs) and 0.25 U HotStartTaq (QIAGEN). All PCR amplifications were carried out on a Thermo Hybaid MBS 0.2S cycler with an initial denaturation of 15 min at 95°C, followed by 40 cycles at 94°C for 45 s, 50°C for 30 s and 72°C for 45 s. Ending with a final extension at 72°C for 10 min, then held at 4°C. Negative controls were included with every PCR reactions as checks for contamination. PCR products were resolved on an ABI PRISM 377 DNA Sequencer (Applied Biosystems). Alleles were scored using GENESCAN version 3.7 (Applied Biosystems) and GeneMarker version 1.71 (SoftGenetics).

### Reliability of genotyping results

We conducted two replicate PCRs for skin samples. Fecal samples were amplified using a modified multiple-tube procedure [39]. In practice, amplification was repeated minimally three times, all heterozygotes were observed in a minimum of two separate reactions and all homozygous showed identical profile of the replicates. Otherwise, we treated the alleles as missing data. Only samples with more than ten loci of allele data were included in statistical analyses. Using GIMLET version 1.3.3 [40], we computed the probability of unrelated individuals or full-sibs bearing an identical multi-locus genotype [*P_ID_* or *P_ID(Sibs)_*]. Fecal samples with identity in all alleles or with only a single mismatch were considered to be deposited by the same individual, and duplicates were removed from the data set. The program MICRO-CHECKER version 2.2.3 [41] was used to check for microsatellite null alleles and scoring errors due to large allele drop-out or stuttering. Rates of genotyping error were calculated following the equations of Broquet and Petit [42].

### Genetic data analyses

Deviations from Hardy–Weinberg Equilibrium (HWE) for each population and genotypic linkage disequilibrium (LD) across all pairs of loci were estimated in GENEPOP version 4.0 [43]. Bonferroni corrections were applied to tests involving multiple comparisons [44]. We used traditional *F*-statistics [45] to assess genetic differentiation, with pairwise *F_ST_* between populations and overall *F_ST_* across the study area. The significance of *F_ST_* values were assessed via 10 000 permutation procedure using FSTAT version 2.9.3.2 [46]. We also calculated the standardized measure of genetic differentiation *F′_ST_* (estimated using *G″_ST_*) to control for the influence of within population heterozygosity [47]–[50], using GenoDive version 2.0b19 [51]. Standard errors of *F′_ST_* estimates were obtained through jackknifing, and the significance of *F′_ST_* values were assessed by permutation test (10 000 permutations) [51]. The genetic diversity within each population, measured as *H_S_* (equal to the expected heterozygosity), were obtained from GenoDive [51].

The program Barrier version 2.2 [52], which implements a computational geometry method and a Monmonier's Maximum-difference algorithm [53], was used to identify the genetic discontinuities within Przewalski's gazelle. This program provides the locations and robustness of the barriers (namely the genetic discontinuities), and visualizes these on a geographical map [52]. In practice, the nine populations of Przewalski's gazelle were connected by a Delaunay triangulation [54] based on each population's average geographic coordinates of sample locations, followed by the application of Monmonier's algorithm with *F_ST_* or *F′_ST_* matrix. To test the robustness of estimated barriers, we used MICROSAT [55] to obtain 100 bootstrap matrices for Barrier significance analysis.

The Bayesian clustering method implemented in software STRUCTURE [56], [57] was used to detect population genetic structure based on individual multilocus genotypes. Ten independent runs of *K* (number of genetic groups) = 1–9 were performed, using correlated allele frequencies [58] and admixture model, with 1 000 000 Markov Chain Monte Carlo (MCMC) repetitions after a 100 000 burn-in period. *K* was identified using the maximum values of *Ln P(D)* (the posterior probability of the data for a given *K*) and *ΔK* (the rate of change in the log probability of data between successive values of *K*) [59]. Bar plots which consist of individual assignment probabilities were applied to cluster the sampling populations into genetic groups.

To assess the genetic variance within and among several possible population groupings, a hierarchical AMOVA (Analysis of Molecular Variance) was performed using Arlequin version 3.11 [60] with 10 000 permutations. We conducted AMOVA analysis on four grouping patterns defined by topography, human settlement, road and railway respectively. These analyses allowed comparing the extent of genetic variance explained by groupings defined by different landscape features. Two additional AMOVA were run on groups identified by inferred genetic discontinuities and genetic structure, respectively.

For migration rate estimation, we used two types of likelihood computation (*L_home_* and *L_home_/L_max_*) implemented in GeneClass version 2.0.h [61] to detect first generation migrants (F_0_). *L_home_* is the likelihood of drawing an individual from the population in which it was sampled, it is appropriate to use when some source populations for immigrants are not sampled [61], [62]. And *L_home_/L_max_* is the ratio of *L_home_* to the greatest likelihood observed in all sampled populations, it is suitable when all source populations are sampled [61], [62]. The likelihood analyses were conducted using a Bayesian method [63], and the probability that an individual is a resident was calculated with a resampling algorithm [62] on 10 000 simulated individuals. To investigate the dispersal pattern of the gazelle, we performed a spatial autocorrelation analysis in GenAlEx 6 [64] which assesses the genetic similarity between pairs of individuals at different geographical distance classes. Because sample sizes were unevenly distributed across distances, variable distance classes were used in the analysis. We ran 1000 random permutations to test the 95% confidence intervals of the null hypothesis, and 1000 bootstraps to estimate 95% confidence intervals for autocorrelation index *r* of each distance class [65]. The significance of *r* was tested by comparing the estimated *r* with the 95% confidence interval about the null hypothesis of a random distribution.

### Landscape genetic analyses

To assess which factors influenced genetic differentiation in Przewalski's gazelle, Mantel tests [66] were applied to test the significance of regression between genetic distance [measured as *F_ST_*/(1−*F_ST_*) and *F′_ST_*/(1−*F′_ST_*), respectively] [67] and geographical distance as well as landscape features. With ArcGIS 9.2 (ESRI), geographical distance was measured as linear Euclidean distances (in km) between populations using the average latitude and longitude of all sample localities within each population (Table 1). For landscape features, a categorical matrix was generated describing whether there were ( = 1) or were not ( = 0) certain type of landscape features between populations. Then, the categorical matrix was used in the Mantel tests [68]. In practice, the categorical matrix was applied to the three main types of anthropogenic landscapes (human settlement, road and railway) and topography (including lake and mountain) (Table 1, 3 and S1). Because geographical distance and landscape features were not independent, partial Mantel tests [69] were used to assess the relative effect of each significant factor inferred in the Mantel tests. All tests were executed using IBD 1.52 [70] with 10 000 permutations to determine statistical significance.

**Table 3:** Matrix of categorical distances of human settlements (upper diagonal) and roads (lower diagonal) between nine populations.

Population	P1	P2	P3	P4	P5	P6	P7	P8	P9
P1	—	1	1	1	1	1	1	1	1
P2	0	—	0	0	1	1	1	1	1
P3	0	0	—	0	1	1	1	1	1
P4	1	1	1	—	1	1	1	1	1
P5	1	1	1	1	—	0	1	1	1
P6	1	1	1	1	0	—	1	1	1
P7	1	1	1	1	1	1	—	1	1
P8	1	1	1	1	1	1	1	—	1
P9	1	1	1	1	1	1	1	1	—

Categorical distances described the presence (1) or absence (0) of the landscape feature between two populations.

To choose the best model interpreting genetic differentiation based on Mantel results, Akaike's information criterion (AIC) which represents how far a tentative model is from the true model was used [71], [72]. We adjusted AIC values to AIC*_c_* values for relatively low number of pairwise comparisons in our data [73]. Lower AIC*_c_* scores imply closer approximation to the true model, and generally, models with AIC*_c_* values greater than 10 compared to the model with the lowest AIC*_c_* value are not supported [72]. Further, we applied Mantel and partial Mantel tests within a causal modeling framework [74] to assess the support for organizational models containing the significant factors inferred in the Mantel tests [using either *F_ST_*/(1−*F_ST_*) or *F′_ST_*/(1−*F′_ST_*)]. This involved computing Mantel and partial Mantel correlation coefficients for each organizational model. Then, the observed correlation coefficients (*P* values) were compared with the expectations of organizational models, and the organizational model with the greatest support was identified.

We also used the hierarchical Bayesian method implemented in GESTE version 2.0 [75] to test the effect of geographical distance, human settlement, road, railway and topography on the genetic differentiation of Przewalski's gazelle. This method estimates *F_ST_* values for each population and relates them to the factors tested using a generalized linear model, and provides posterior probabilities for each model using a reversible jump Markov Chain Monte Carlo (MCMC) approach. Three independent replicates were executed with default parameter values of the program to ensure consistency of results.

## Results

### Reliability of genotyping results

We collected 28 skin samples and 182 fresh fecal samples from the field. We amplified 25 skin and 161 fecal samples with more than ten loci. The unbiased *P_ID_* of 3.48×10^−9^ and *P_ID(Sibs)_* of 2.63×10^−4^ indicates high reliability of the 13 microsatellite markers for distinguishing fecal samples from the same individual. After removing duplicate fecal samples, we obtained genotype data for 169 individuals (P1, 24; P2, 38; P3, 32; P4, 3; P5, 8; P6, 19; P7, 3; P8, 21; P9, 21). The total genotyping error rate was 0.328%, indicating high reliability of the data. MICRO-CHECKER did not detect the presence of null alleles or scoring errors.

### HWE and LD

At the 0.05 significant level, all microsatellite loci were in HWE for each population and the entire sample. Thirty seven out of 702 loci pairs for each population (P1, 2; P2, 5; P3, 10; P5, 2; P6, 6; P8, 3; P9, 9) and seven out of 78 loci pairs for the entire sample showed significant LD. After Bonferroni correction, genotypic linkage disequilibrium was not significant.

### Population genetic differentiation

**Figure 2:**
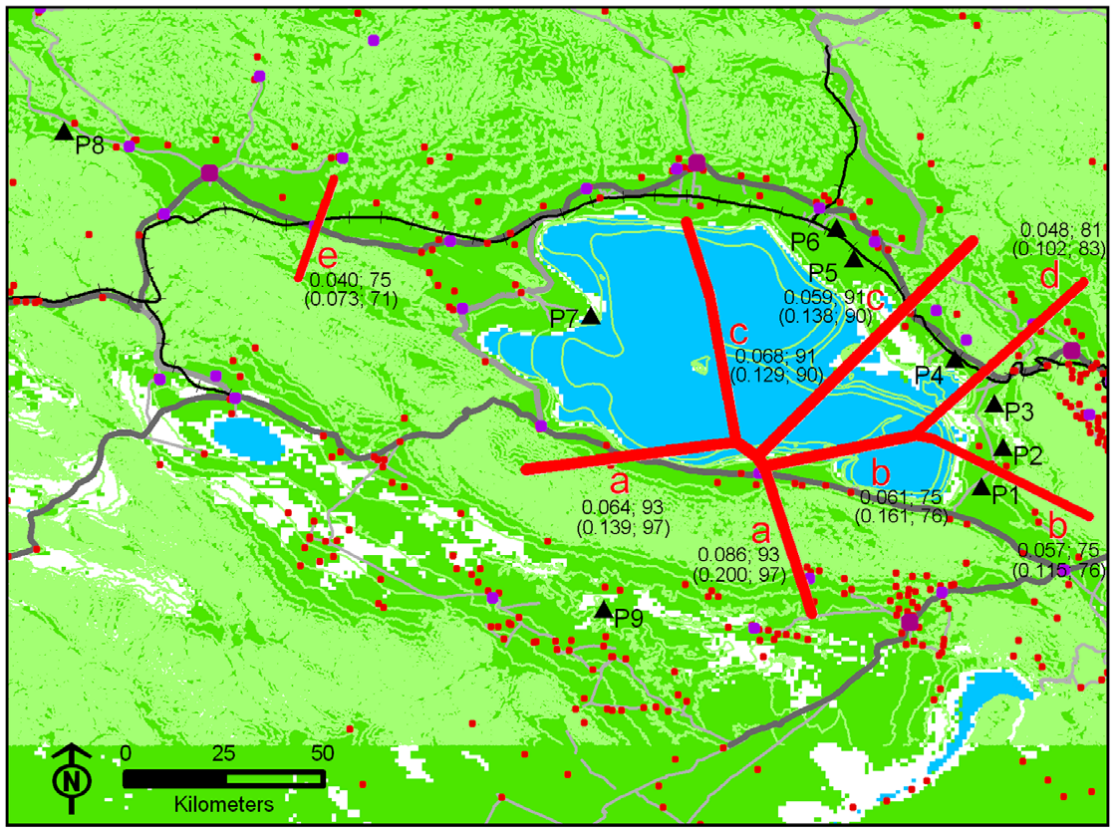
Barrier analysis detected five genetic discontinuities (red lines, a–e) among nine populations of Przewalski's gazelle. These barriers separate the nine populations into seven groups: P1, P2–P3, P4, P5–P6, P7, P8, P9. The *F_ST_* and bootstrap values are shown near the barriers with the *F′_ST_* and bootstrap values showing in the brackets.

**Figure 3:**
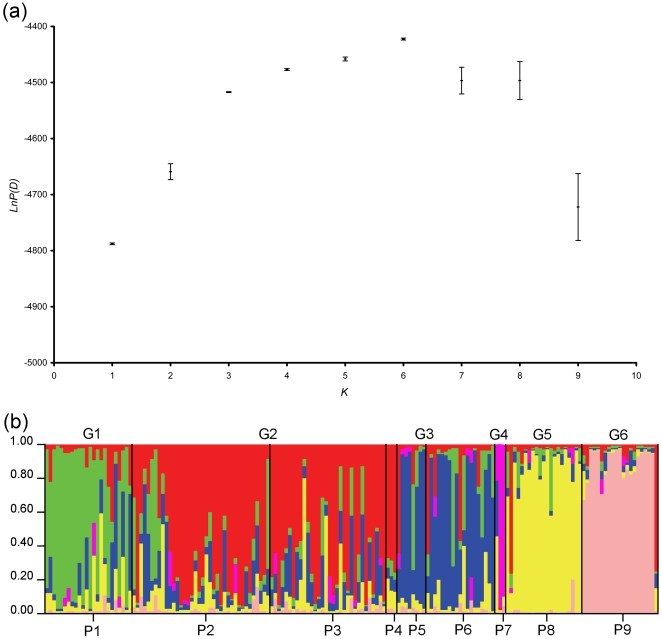
Output of Structure analysis based on *Ln P(D)* values. (a) Average values of *Ln P(D)* show that the highest log likelihood occurs at *K* = 6 genetic groups. (b) Bar plot of six genetic groups. The sampling populations for individuals are shown as P1–P9, and the genetic groups assigned (G1–G6) are shown above: G1 including P1–green; G2 including P2, P3 and P4–red; G3 including P5 and P6–blue; G4 including P7–purple; G5 including P8–yellow; G6 including P9–pink.

The global *F_ST_* and *F′_ST_* across the study area was 0.072 (*P*<0.001) and 0.147 (*P*<0.001) respectively. Pairwise *F_ST_* between sampling populations ranged from 0.0002 to 0.1152, and the range of pairwise *F′_ST_* were from 0.000 to 0.257 (Table 4). From both measures (*F_ST_* or *F′_ST_*), the results indicated significant genetic differentiation between the population pairs except P2–P3 pair, P5–P6 pair, and pairs involving P4 or P7 (Table 4). Within-population genetic diversity (*H_S_*) ranged from 0.462 in P3 to 0.641 in P7 (Table S2). The program Barrier identified five genetic discontinuities (a–e) with high bootstrap support regardless which differentiation measure (*F_ST_* or *F′_ST_*) was used, which separated the nine populations of Przewalski's gazelle into seven groups: P1, P2–P3, P4, P5–P6, P7, P8, P9 (Figure 2). In the Structure analysis, average *Ln P(D)* maximized at *K* = 6 genetic groups consisting of: G1 (P1), G2 (P2–P3–P4), G3 (P5–P6), G4 (P7), G5 (P8) and G6 (P9) (Figure 3), whereas the highest value of average *ΔK* emerged at *K* = 3 genetic groups containing: G′1 (P1, P5, P6, P7, P8), G′2 (P2, P3, P4) and G′3 (P9) (Figure S1). Because the main incongruence was that whether G′1 was subdivided, we re-ran STRUCTURE using only the data of G′1 to test for further subdivision. The results showed that four genetic groups (the same as G1, G3, G4, G5) did exist within G′1 (Figure S2). Overall, Structure analyses indicated that there were most likely six genetic groups (G1–G6) within Przewalski's gazelle. These genetic groups were the same as AMOVA grouping defined by human settlement. The AMOVA results revealed a significant proportion of genetic variance among groups in all cases, however, the proportion of variance among groups defined by human settlement or genetic structure from Structure (7.68%, *P*<0.001) and genetic discontinuities from Barrier (7.90%, *P*<0.001) were much higher (Table 5). The groupings defined by topography, road and railway also showed significant genetic variance among populations within groups (Table 5).

**Table 4:** Matrix of pairwise *F_ST_* estimates and their statistical significance (upper diagonal), and matrix of the standardized genetic differentiation *F′_ST_* and standard error as well as statistical significance (lower diagonal) between nine populations.

Population	P1	P2	P3	P4	P5	P6	P7	P8	P9
P1	—	0.0565*	0.0694*	0.0611	0.0563*	0.0499*	0.0126	0.0707*	0.0862*
P2	0.115±0.028*	—	0.0002	0.0418	0.1044*	0.0621*	0.0909	0.0825*	0.1152*
P3	0.141±0.033*	0.000±0.008	—	0.0482	0.0952*	0.0529*	0.1068	0.0956*	0.1138*
P4	0.161±0.083	0.090±0.088	0.102±0.122	—	0.0589	0.0679	0.0255	0.0431	0.1055
P5	0.129±0.071*	0.199±0.062*	0.179±0.058*	0.138±0.072	—	0.0085	0.0387	0.1088*	0.0873*
P6	0.112±0.050*	0.122±0.043*	0.103±0.041*	0.159±0.107	0.018±0.024	—	0.0676	0.0928*	0.0841*
P7	0.011±0.084	0.149±0.108	0.180±0.129	0.060±0.125	0.072±0.093	0.129±0.112	—	0.0404	0.0642
P8	0.162±0.039*	0.165±0.053*	0.190±0.058*	0.114±0.057	0.240±0.068*	0.203±0.051*	0.073±0.069	—	0.1036*
P9	0.200±0.050*	0.232±0.059*	0.228±0.051*	0.257±0.078	0.198±0.049*	0.186±0.037*	0.139±0.086	0.235±0.062*	—

*P<0.0014 [significant level of *P* (0.05) was adjusted for multiple comparisons].

**Table 5:** Analysis of Molecular Variance (AMOVA) on six grouping patterns.

Groups defined	Components	Percentage of variation
Three groups according to topography:	Among groups	4.92***
[P1–P6][P7, P8][P9]	Among pops within groups	4.53***
	Within pops	90.55***
Six groups according to human settlement:	Among groups	7.68***
[P1][P2–P4][P5, P6] [P7] [P8] [P9]	Among pops within groups	0.41
	Within pops	91.91***
Six groups according to road:	Among groups	5.46***
[P1–P3][P4][P5, P6] [P7] [P8] [P9]	Among pops within groups	2.99***
	Within pops	91.55***
Three groups according to railway:	Among groups	0.84***
[P1–P5, P7, P9][P6][P8]	Among pops within groups	6.73***
	Within pops	92.43
Seven groups according to gentic discontinuities:	Among groups	7.90***
[P1][P2, P3] [P4] [P5, P6] [P7] [P8] [P9]	Among pops within groups	0.09
	Within pops	92.01***
Six groups according to gentic structure:	Among groups	7.68***
[P1][P2–P4][P5, P6] [P7] [P8] [P9]	Among pops within groups	0.41
	Within pops	91.91***

***P<0.001.

### Dispersal pattern

**Figure 4:**
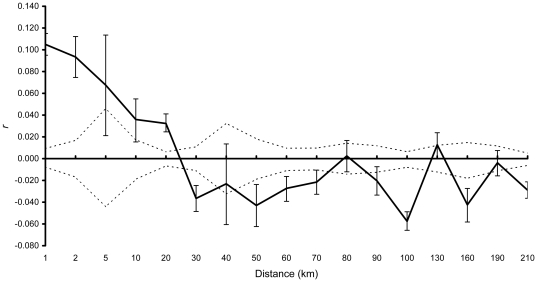
Spatial autocorrelation analysis. Correlogram plots of the genetic autocorrelation index *r* (black line) as a function of geographical distance for entire sample. Two dotted lines indicate the 95% confidence interval about the null hypothesis of a random distribution of the gazelles. The error bars about *r* indicate 95% confidence interval determined by bootstrapping.

In the GeneClass analysis, we detected four first generation migrants based on *L_home_/L_max_*. They were Hudong4 (in P2) from P3, Ketu10 (in P3) from P2, Shadao2 (in P4) from P3 and HerG8 (in P6) from P5. Using *L_home_*, in addition to the same four F_0_ being identified, another first generation migrant (Hudong7) in P2 was detected from P1. Spatial autocorrelation analysis displayed positive and significant *r* values for the first five distance classes (1, 2, 5, 10 and 20 km), and the highest *r* value was observed within two kilometers (*r* was around 0.1, *P* = 0.001). The correlogram flattened out after 20 km, then *r* values remained slightly negative up to 80 km, followed by oscillation (Figure 4).

### Landscape genetic analyses

**Figure 5:**
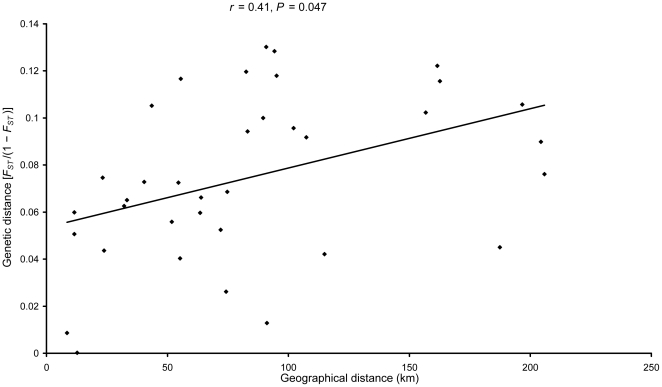
Euclidean geographical distance (km) and genetic distance [*F_ST_*/(1−*F_ST_*)] are positively correlated (*r* = 0.41, *P* = 0.047) in Mantel test.

In Mantel tests, the results from two genetic distance measures [*F_ST_*/(1−*F_ST_*) or *F′_ST_*/(1−*F′_ST_*)] were largely consistent. Significant positive associations were found between genetic differentiation and human settlement, road or topography, but genetic differentiation was not significantly correlated with the presence of railway (Table 6). When using *F_ST_*/(1−*F_ST_*), Mantel tests also indicated that 17.04% of the genetic differentiation could be explained by IBD (isolation by distance) (Figure 5). Regardless of the genetic distance measure, the results of partial Mantel tests showed significant correlation between genetic differentiation and human settlement (Table 6), and AIC*_c_* calculations demonstrated that the models concerning human settlement had the lowest AIC*_c_* values both in Mantel and partial Mantel tests, thus they were superior to other models (Table 6). From causal modeling analysis with either genetic distance measure, only the isolation by human settlement model was fully supported by all statistical expectations (Table 7 and S3). This model predicts that genetic differentiation is mainly attributable to the effect of human settlement with no significant independent relationships with geographical distance, road or topography. GESTE generated 32 models and gave consistent results across three replicates. The null model which excluded all tested factors was inferred as the best model because it had the highest posterior probability, and the values of other models were very low (Table 8), suggesting little effect of tested factors on observed genetic differentiation. However, the estimate of *σ^2^* for the null model, a measure of model fit, was very high with the upper bound of the HPDI over 1 [0.45, HPDI (0.11; 1.04)], indicating that GESTE failed to identify the true model [75].

**Table 6:** Correlations between genetic distance [*F_ST_*/(1−*F_ST_*) and *F′_ST_*/(1−*F′_ST_*), respectively] and geographical distance as well as human settlement, road, railway, topography as measured by Mantel tests, partial Mantel tests and AIC calculations.

*Mantel tests and partial Mantel tests*	*r*	*R^2^ (%)*	*P*	AIC*_c_*
Geographical distance	0.41 (0.44)	17.04 (19.74)	**0.047** (0.081)	−247.21 (−182.29)
Human settlement	0.50 (0.48)	25.37 (23.11)	**0.000** (**0.002**)	−251.05 (−183.83)
Road	0.40 (0.40)	16.01 (15.82)	**0.014** (**0.012**)	−246.78 (−180.57)
Railway	0.00 (0.03)	0.00 (0.11)	0.474 (0.371)	−240.50 (−174.41)
Topography	0.42 (0.43)	17.84 (18.33)	**0.013** (**0.015**)	−247.58 (−181.66)
Geographical distance (controlling for human settlement)	0.25 (0.30)	9.79 (12.83)	0.133 (0.126)	−241.97 (−177.07)
Geographical distance (controlling for road)	0.29 (0.33)	12.12 (14.84)	0.115 (0.121)	−242.91 (−177.91)
Geographical distance (controlling for topography)	0.19 (0.23)	9.65 (12.66)	0.189 (0.166)	−241.91 (−177.00)
Human settlement (controlling for geographical distance)	0.39 (0.36)	20.12 (17.01)	**0.014** (**0.021**)	−246.33 (−178.84)
Human settlement (controlling for road)	0.40 (0.37)	20.47 (18.24)	**0.009** (**0.015**)	−246.50 (−179.38)
Human settlement (controlling for topography)	0.40 (0.38)	20.10 (17.75)	**0.013** (**0.014**)	−246.32 (−179.16)
Road (controlling for geographical distance)	0.27 (0.25)	10.84 (9.97)	0.069 (0.083)	−242.39 (−175.91)
Road (controlling for human settlement)	0.23 (0.24)	8.90 (9.22)	0.110 (0.102)	−241.61 (−175.61)
Road (controlling for topography)	0.28 (0.28)	11.06 (10.77)	0.054 (0.053)	−242.47 (−176.23)
Topography (controlling for geographical distance)	0.21 (0.19)	11.13 (10.04)	0.166 (0.217)	−242.50 (−175.94)
Topography (controlling for human settlement)	0.28 (0.30)	11.18 (12.08)	0.084 (0.093)	−242.52 (−176.76)
Topography (controlling for road)	0.31 (0.32)	13.23 (13.75)	0.052 (0.069)	−243.36 (−177.45)

Correlation coefficient (*r*), determination of genetic distance [*R^2^ (%)*], significance index (*P*) and Akaike's information criterion value (AIC*_c_*) for each test are presented. *P*<0.05 indicates statistical significant (values in bold type). The results of *F′_ST_*/(1−*F′_ST_*) are shown in the brackets.

**Table 7:** Four most probable organizational models when using the genetic distance *F_ST_*/(1−*F_ST_*) in Mantel tests.

Organizational model	Expectation		*P* value	Support rate
Model 1 isolation by human settlement	HG. D	S	**0.014**	1.00
	HG. R	S	**0.009**	
	HG. T	S	**0.013**	
	DG. H	NS	**0.133**	
	RG. H	NS	**0.110**	
	TG. H	NS	**0.084**	
Model 2 isolation by distance and human settlement	DG. H	S	0.133	0.70
	DG. R	S	0.115	
	DG. T	S	0.189	
	HG. D	S	**0.014**	
	HG. R	S	**0.009**	
	HG. T	S	**0.013**	
	RG. D	NS	**0.069**	
	RG. H	NS	**0.110**	
	TG. D	NS	**0.166**	
	TG. H	NS	**0.084**	
Model 3 isolation by human settlement and road	HG. D	S	**0.014**	0.70
	HG. R	S	**0.009**	
	HG. T	S	**0.013**	
	RG. D	S	0.069	
	RG. H	S	0.110	
	RG. T	S	0.054	
	DG. H	NS	**0.133**	
	DG. R	NS	**0.115**	
	TG. H	NS	**0.084**	
	TG. R	NS	**0.052**	
Model 4 isolation by human settlement and topography	HG. D	S	**0.014**	0.70
	HG. R	S	**0.009**	
	HG. T	S	**0.013**	
	TG. D	S	0.166	
	TG. H	S	0.084	
	TG. R	S	0.052	
	DG. H	NS	**0.133**	
	DG. T	NS	**0.189**	
	RG. H	NS	**0.110**	
	RG. T	NS	**0.054**	

D = distance, H = human settlement, R = road, T = topography, G = genetic distance. The period in the expectation abbreviations separate the covariate matrix from the two primary matrices. For example, DG. H indicates a partial Mantel test between the geographical distance and genetic matrices, with the human settlement matrix partialed out. Boldface indicates that the *P* value matches the expectations of the model. S = significant, NS = not significant, *P*<0.05 indicates significant. Other eleven models with support rate ≤0.5 were not presented.

**Table 8:** Posterior probabilities of the seven most probable models explaining genetic differentiation of Przewalski's gazelle populations, as determined by the program GESTE.

Model	Factors included	Posterior probability
1	Null	0.666
2	Human settlement	0.064
3	Road	0.056
4	Topography	0.051
5	Geographical distance	0.049
6	Railway	0.046
7	Human settlement, topography	0.011

The posterior probability was averaged over the three replicates. Other 25 models with posterior probability <0.01 were not exhibited.

## Discussion

The *F*-statistics and *F′_ST_* analyses revealed significant genetic differentiation in the entire sample and between most populations (Table 4). The results are within our expectation given that the distribution of Przewalski's gazelle has been highly fragmented by anthropogenic landscapes (Figure 1). For population pairs with no significant differentiation, comparisons involving P4 or P7 could be due to its small sample size. P2 and P3 may exchange individuals via barrier-free desert. P5 and P6 are separated by the Qinghai-Tibet railway, yet a bridge with underpasses is present and could allow the migration of gazelles. Although *F′_ST_* presented larger global and pairwise values than *F*-statistics analysis (Table 4), suggesting a higher level of actual genetic differentiation than indicated by *F*-statistics, the differentiation pattern inferred from *F′_ST_* and *F*-statistics results were largely consistent. For example, *F′_ST_* and *F*-statistics indicated significant genetic differentiation between the same population pairs, and they ranked the pairwise differentiation similarly (Table 4). In the Barrier analysis using either *F_ST_* or *F′_ST_* matrices, we found five genetic discontinuities and seven groups (Figure 2) which are largely in accordance with the observed pattern of genetic differentiation. Genetic structure analysis using Bayesian clustering method detected six genetic groups (Figure 3) nearly identical to the groups inferred by Barrier (except P4), corroborating the results from *F*-statistics, *F′_ST_* and Barrier analysis. The AMOVA results showed that the grouping patterns according to genetic discontinuities, genetic structure and human settlement explained relatively high proportion of genetic variance among groups and revealed small and nonsignificant among populations within groups (Table 5), thus these groupings seem to be rational for characterizing the distribution pattern of genetic variance. Previous research on Przewalski's gazelle using mtDNA control region sequences revealed a population genetic structure (Yuanzhe – P1 in this study, Hudong-Ketu – P2 and P3 here, Shadao-Gahai – P4 here, and Bird Island – P7 here) consistent with the results of genetic differentiation in this study [26].

GeneClass analysis found five first generation migrants between four pairs of neighbouring populations (P1–P2; P2–P3; P3–P4; P5–P6), indicating low migration rate and restricted dispersal in Przewalski's gazelle. Moreover, the results from GeneClass also corroborated the observed pattern of genetic differentiation, because four F_0_ were found between the populations not differentiated. Spatial autocorrelation analysis detected positive autocorrelation at shorter distances as predicted under a restricted dispersal model (Figure 4), suggesting that dispersal events of the gazelle were confined to small geographical scale and most likely to occur between neighbouring populations. The highest autocorrelation index *r* was found within two kilometers which is approximately equal to the population home range of the gazelle [25], implying that individuals within the same population are closely related. Overall, the results indicated a restricted dispersal pattern of Przewalski's gazelle, congruent with the significant genetic differentiation observed.

Several previous studies have revealed significant genetic effects of anthropogenic landscapes on wild ungulates, such as desert bighorn sheep *Ovis canadensis nelsoni* [5], European roe deer *Capreolus capreolus* [76] and Scottish highland red deer *Cervus elaphus* [77]. In our research, landscape genetic analysis also yielded evidence for significant genetic influences from anthropogenic landscape features on Przewalski's gazelle. Mantel tests found that human settlement, road and topography are significant factors responsible for current population genetic differentiation regardless of the measure of genetic distance [*F_ST_*/(1−*F_ST_*) or *F′_ST_*/(1−*F′_ST_*)] (Table 6). Geographical distance was also inferred as a significant factor in Mantel tests when genetic distance was measured as *F_ST_*/(1−*F_ST_*) (Figure 5). The results of partial Mantel tests, AIC*_c_* calculations and causal modeling analysis with both genetic distance measures further demonstrated that human settlement was the main factor (Table 6, 7 and S3). The AMOVA analyses showed that the grouping defined by human settlement interpreted much higher proportion of genetic variance among groups than those defined by other landscapes (Table 5), thus human settlement appears to be the most important landscape feature among those tested determining the distribution pattern of genetic variance. GESTE analysis did not provide reliable results for identifying the effect of tested factors on observed genetic differentiation, probably because the number of populations in our data was small. Foll and Gaggiotti have suggested that the analytical power of GESTE can be limited by the number of populations analysed (<20 populations), and the method may fail to identify the true model with less than ten populations [75].

The primary role of human settlement could be corroborated by following observations. First, P1, P2 and P3 provide a good example showing the large genetic effect of human settlement (Figure 1). These populations are located on the same side of a road, with a nearly equal interval of 12 km. Several villages separate P1 from P2 and no barrier exists between P2 and P3. In 2this study, we found that P1 and P2 had significant genetic differentiation (Table 4) and the villages between them coincided with the location of inferred genetic discontinuity b (Figure 2), but P2 and P3 presented no differentiation (Table 4). We can see that, even over a short distance of 12 km, human settlements seem to have significantly influenced population genetic differentiation of the species. Second, the people living in this area are nomads. They once hunted gazelles for food in winter, and their livestock compete with the gazelle for food resources [78], [79]. Moreover, Przewalski's gazelle are extremely vigilant towards human activity and escape swiftly when people approach them within 500 m [21], and they have shifted their peak foraging time to the mornings and evenings to avoid livestock grazing and human disturbance [20]. Therefore, the physical barrier of human settlement may be amplified by associated human activities such as hunting and livestock grazing. Third, although human population density is very low throughout the study area (four persons per km^2^), it is much higher within human settlement areas (about 1000 to 1600 persons per km^2^).

Besides human settlement, attention should also be given to the effect of geographical distance, road and topography. As a restricted dispersal pattern was revealed in Przewalski's gazelle, geographical distance seems to be an important influencing factor for distant populations. The study area is fragmented by roads (Figure 1), and a recent study suggested that roads around Qinghai Lake changed the diurnal activity of the gazelle [80]. Provided that the traffic volume continues to increase along with the development of economy, roads would certainly exert larger influence on the gazelle. Based on our field research experience, the gazelle is unable to traverse the Qinghai Lake or mountain ridge, hence topography appears to play an important role on the distribution of and gene flow between populations of Przewalski's gazelle.

The railway showed no influence on genetic differentiation, probably because of its short history, limited distribution and infrequent traffic (Table 2). Moreover, the bridges and underpasses along the railway are well designed and most likely allow Przewalski's gazelle to safely cross this infrastructure, for example, between P5 and P6. Across the study area, there are other anthropogenic landscape features (e.g. grassland fence) which were not considered here because of very short history (constructed in the last 15 years) and the ability of the gazelle to cross them (e.g. Przewalski's gazelle frequently jump over the grassland fence).

Based on our findings in this study, we propose the following measures to conserve Przewalski's gazelle. First, since human settlement has large effect on Przewalski's gazelle, measures mitigating its negative influence should be carried out. One sensible strategy is collaboration with local community to protect the gazelle, through conservation communication et al. This proposal is probably realistic, given that local people are in favor of protecting the gazelle [81]. Second, according to the identified first generation migrants and the restricted dispersal pattern in the gazelle, suitable habitat for Przewalski's gazelle between the six populations (P1–P6) with short geographical distance should be protected and restored to facilitate the potential migration. Third, besides human settlement, the effect of other anthropogenic landscape features, such as road, railway and grassland fence, should not be neglected and should be minimized through the construction of wildlife underpass and lowering of the fence.

In conclusion, our results indicated significant genetic differentiation, five genetic discontinuities, six genetic groups and restricted dispersal pattern in Przewalski's gazelle, and showed that, under realistic circumstance with compound effect, IBD, topography and some anthropogenic landscape features (human settlements and roads) have been affecting the process of population genetic differentiation. Furthermore, using multiple methods, we demonstrated that human settlement is the main factor shaping current genetic differentiation among those tested. In conservation context, we propose several conservation strategies, such as habitat protection and restoration between close populations, and collaboration with local community. It is possible that other factors, biotic as well as abiotic, may have contributed to the genetic structure of this species, although the analyses presented here seem to point clearly at human settlements being the primary agent responsible for differentiation. To our knowledge, this is the first study assessing the genetic effect of anthropogenic landscape features on wild ungulate in an alpine grassland ecosystem on the Qinghai-Tibet Plateau. Because Przewalski's gazelle is a flagship species [18], our research provides valuable ideas for the management of other wild animals such as larger mammals across the region.

## Supporting Information

Figure S1 **Output of Structure analysis according to** ***ΔK*** **values.** (a) Plot of *ΔK* indicates that there are most likely three genetic groups. (b) Bar plot of three genetic groups. The sampling populations for individuals are shown as P1–P9, and the genetic groups assigned (G′1–G′3) are shown above: G′1 including P1, P5, P6, P7 and P8–red; G′2 including P2, P3 and P4–blue; G′3 including P9–green.
(TIF)

Figure S2 **Output of Structure analysis using the data of G′1.** (a) Average values of *Ln P(D)* show that the highest log likelihood occurs at *K* = 4 genetic groups. (b) Plot of *ΔK* indicates that there are most likely four genetic groups. (c) Bar plot of four genetic groups. The sampling populations for individuals are shown as P1, P5, P6, P7 and P8, and the genetic groups assigned are shown above: G1 including P1–red; G3 including P5 and P6–blue; G4 including P7–yellow; G5 including P8–green.
(TIF)

Table S1 Matrix of categorical distance of railway (lower diagonal) between nine populations.
(DOC)

Table S2 The sample size and genetic diversity within each population.
(DOC)

Table S3 Evaluation of seven organizational models when using the genetic distance *F′_ST_*/(1−*F′_ST_*) in Mantel tests.
(DOC)

## References

[1] Fisher RA, Ford EB (1947). The spread of a gene in natural conditions in a colony of moth *Panaxia dominula*.. L Heredity.

[2] Hitchings SP, Beebee TJC (1997). Genetic substructuring as a result of barriers to gene flow in urban *Rana temporaria* (common frog) populations: implications for biodiversity conservation.. Heredity.

[3] Gerlach G, Musolf K (2000). Fragmentation of landscape as a cause for genetic subdivision in bank voles.. Conserv Biol.

[4] Keller I, Largiadèr CR (2003). Recent habitat fragmentation caused by major roads leads to reduction of gene flow and loss of genetic variability in ground beetles.. Proc R Soc Lond, Ser B: Biol Sci.

[5] Epps CW, Palsboll PJ, Wehausen JD, Roderick GK, Ramey RR (2005). Highways block gene flow and cause a rapid decline in genetic diversity of desert bighorn sheep.. Ecol Lett.

[6] Manel S, Schwartz MK, Luikart G, Taberlet P (2003). Landscape genetics: combining landscape ecology and population genetics.. Trends Ecol Evol.

[7] Keyghobadi N, Roland J, Strobeck C (1999). Influence of landscape on the population genetic structure of the alpine butterfly *Parnassius smintheus* (Papilionidae).. Mol Ecol.

[8] Funk WC, Blouin MS, Corn PS, Maxell BA, Pilliod DS (2005). Population structure of Columbia spotted frogs (*Rana luteiventris*) is strongly affected by the landscape.. Mol Ecol.

[9] Noel S, Ouellet M, Galois P, Lapointe FJ (2007). Impact of urban fragmentation on the genetic structure of the eastern red-backed salamander.. Conserv Genet.

[10] Liu Z, Ren B, Wu R, Zhao L, Hao Y (2009). The effect of landscape features on population genetic structure in Yunnan snub-nosed monkeys (*Rhinopithecus bieti*) implies an anthropogenic genetic discontinuity.. Mol Ecol.

[11] Keller I, Nentwig W, Largiadèr CR (2004). Recent habitat fragmentation due to roads can lead to significant genetic differentiation in an abundant flightless ground beetle.. Mol Ecol.

[12] Johnson MS, Black R (1995). Neighbourhood size and the importance of barriers to gene flow in an intertidal snail.. Heredity.

[13] Johannesen J, Veith M, Seitz A (1996). Population genetic structure of the butterfly *Melitaea didyma* (Nymphalidae) along a northern distribution range border.. Mol Ecol.

[14] Williams BL, Brawn JD, Paige KN (2003). Landscape scale genetic effects of habitat fragmentation on a high gene flow species: *Speyeria idalia* (Nymphalidae).. Mol Ecol.

[15] Luoy D, Habel JC, Schmitt T, Assmann T, Meyer M (2007). Strongly diverging population genetic patterns of three skipper species: the role of habitat fragmentation and dispersal ability.. Conserv Genet.

[16] Mallon DP, Jiang Z (2009). Grazers on the plains: challenges and prospects for large herbivores in Central Asia.. J Appl Ecol.

[17] Mallon DP, Kingswood SC (2001). Antelopes. Part 4: North Africa, the Middle East, and Asia. Global Survey and Regional Action Plans. SSC Antelope Specialist Group..

[18] Jiang Z, Li D, Wang Z, Zhu S, Wei W (2001). Population structure of the Przewalski's gazelle around the Qinghai Lake, China.. Acta Zool Sin.

[19] Jiang Z, Feng Z, Wang Z, Chen L, Cai P (1995). Historical and current distributions of Przewalski's gazelle.. Acta Theriol Sin.

[20] Jiang Z, Li D, Wang Z (2000). Population declines of Przewalski's gazelle around Qinghai Lake, China.. Oryx.

[21] Jiang Z (2004). Przewalski's gazelle.

[22] Ye R, Cai P, Peng M, Lu X, Ma S (2006). The investigation about distribution and population size of Przewalski's gazelle (*Procapra przewalskii*) in Qinghai province, China.. Acta Theriol Sin.

[23] IUCN SSC Antelope Specialist Group (2008). *Procapra przewalskii*.. IUCN 2009, IUCN Red List of Threatened Species, Version 2009.1.

[24] Li D, Jiang Z, Wang Z (1999). Activity patterns and habitat selection of the Przewalski's gazelle (*Procapra Przewalskii*) in the Qinghai Lake region.. Acta Theriol Sin.

[25] Li Z (2008). Competition and coexistence mechanisms of sympatric Przewalski's gazelle and Tibetan gazelle in upper Buha river, Qinghai-Tibet plateau..

[26] Lei R, Hu Z, Jiang Z, Yang W (2003). Phylogeography and genetic diversity of the critically endangered Przewalski's gazelle.. Anim Conserv.

[27] Gonghe County Chronicles Compilation Committee (1991). Gonghe county chronicles.

[28] Haiyan County Chronicles Compilation Committee (1994). Haiyan county chronicles.

[29] Tianjun County Chronicles Compilation Committee (1995). Tianjun county chronicles.

[30] Gangcha County Chronicles Compilation Committee (1998). Gangcha county chronicles.

[31] Fu X (2000). An analysis of urbanization and its dynamical mechanism in Qinghai-Tibet plateau.. J Nat Resour.

[32] Gao X, Wang Y, Feng Y, Wang J, Ma A (2002). Study on land use change and its influence on eco-environment in Qinghai Lake region.. Remote Sens Technol Appl.

[33] Liu C (2005). Analysis on the history and present condition of population urbanization in Qinghai province and the future concept.. Nationalities Res in Qinghai.

[34] Chen K, Li S, Zhou Q, Duo H, Chen Q (2008). Analyzing dynamics ecosystem service values based on variations of landscape patterns in Qinghai Lake area in recent 25 Years.. Resour Sci.

[35] Maudet C, Luikart G, Dubray D, Von Hardenberg A, Taberlet P (2004). Low genotyping error rates in wild ungulate faeces sampled in winter.. Mol Ecol Notes.

[36] Sambrook J, Fritsch EF, Maniatis T (1989). Molecular Cloning: a Laboratory Manual, 2nd ed.

[37] Slate J, Coltman DW, Goodman SJ, MacLean I, Pemberton JM (1998). Bovine microsatellite loci are highly conserved in red deer (*Cervus elaphus*), sika deer (*Cervus nippon*) and Soay sheep (*Ovis aries*).. Anim Genet.

[38] Slate J, Van Stijn TC, Anderson RM, Mary McEwan K, Maqbool NJ (2002). A deer (subfamily Cervinae) genetic linkage map and the evolution of ruminant genomes.. Genetics.

[39] Taberlet P, Griffin S, Goossens B, Questiau S, Manceau V (1996). Reliable genotyping of samples with very low DNA quantities using PCR.. Nucleic Acids Res.

[40] Valière N (2002). GIMLET: a computer program for analysing genetic individual identification data.. Mol Ecol Notes.

[41] Van Oosterhout C, Hutchinson WF, Wills DPM, Shipley P (2004). MICRO-CHECKER: software for identifying and correcting genotyping errors in microsatellite data.. Mol Ecol Notes.

[42] Broquet T, Petit E (2004). Quantifying genotyping errors in noninvasive population genetics.. Mol Ecol.

[43] Raymond M, Rousset F (1995). GENEPOP (version 1.2): Population genetics software for exact tests and ecumenicism.. J Hered.

[44] Rice W (1989). Analyzing tables of statistical tests.. Evolution.

[45] Wright S (1978). Evolution and the genetics of populations, Vol 4.

[46] Goudet J (2002). FSTAT, a program to estimate and test gene diversities and fixation indices, version 2.9.3.2.. http://www2.unil.ch/popgen/softwares/fstat.htm.

[47] Hedrick PW (2005). A standardized genetic differentiation measure.. Evolution.

[48] Jost L (2008). *G_ST_* and its relatives do not measure differentiation.. Mol Ecol.

[49] Heller R, Siegismund HR (2009). Relationship between three measures of genetic differentiation *G_ST_*, *D_EST_* and *G′_ST_*: how wrong have we been?. Mol Ecol.

[50] Meirmans PG, Hedrick PW (2011). Assessing population structure: *F_ST_* and related measures.. Mol Ecol Resour.

[51] Meirmans PG, Van Tienderen PH (2004). GENOTYPE and GENODIVE: two programs for the analysis of genetic diversity of asexual organisms.. Mol Ecol Notes.

[52] Manni F, Guérard E, Heyer E (2004). Geographic pattern of (genetic, morphologic, linguistic) variation: how barriers can be detected by using ‘Monmonier's algorithm’.. Hum Biol.

[53] Monmonier MS (1973). Maximum-difference barriers: an alternative numerical regionalization method.. Geogr Anal.

[54] Brassel KE, Reif D (1979). A procedure to generate Thiessen polygons.. Geogr Anal.

[55] Minch E, Ruiz-Linares A, Goldstein DB, Feldman MW, Cavalli-Sforza LL (1997). MICROSAT: a computer program for calculating various statistics on microsatellite allele data.

[56] Pritchard JK, Stephens M, Donnelly P (2000). Inference of population structure using multilocus genotype data.. Genetics.

[57] Pritchard JK, Wen X, Falush D (2007). STRUCTURE version 2.2..

[58] Falush D, Stephens M, Pritchard JK (2003). Inference of population structure: extensions to linked loci and correlated allele frequencies.. Genetics.

[59] Evanno G, Regnaut S, Goudet J (2005). Detecting the number of clusters of individuals using the software STRUCTURE: a simulation study.. Mol Ecol.

[60] Excoffier L, Laval G, Schneider S (2005). Arlequin (version 3.0): an integrated software package for population genetics data analysis.. Evol Bioinform online.

[61] Piry S, Alapetite A, Cornuet JM, Paetkau D, Baudouin L (2004). GeneClass2: a software for genetic assignment and first-generation migrant detection.. J Hered.

[62] Paetkau D, Slade R, Burden M, Estoup A (2004). Genetic assignment methods for the direct, real-time estimation of migration rate: a simulation-based exploration of accuracy and power.. Mol Ecol.

[63] Rannala B, Mountain JL (1997). Detecting immigration by using multilocus genotypes.. Proc Natl Acad Sci U S A.

[64] Peakall R, Smouse PE (2006). GenAlEx 6: genetic analysis in Excel, population genetic software for teaching and research.. Mol Ecol Notes.

[65] Peakall R, Ruibal M, Lindenmayer DB (2003). Spatial autocorrelation analysis offers new insights into gene flow in the Australian bush rat, *Rattus fuscipes*.. Evolution.

[66] Mantel N (1967). The detection of disease clustering and a generalized regression approach.. Cancer Res.

[67] Rousset F (1997). Genetic differentiation and estimation of gene flow from F-statistics under isolation by distance.. Genetics.

[68] Lampert KP, Rand AS, Mueller UG, Ryan MJ (2003). Fine-scale genetic pattern and evidence for sex-biased dispersal in the túngara frog, *Physalaemus pustulosus*.. Mol Ecol.

[69] Smouse PE, Long JC, Sokal RR (1986). Multiple regression and correlation extensions of the Mantel test of matrix correspondence.. System Zool.

[70] Bohonak AJ (2002). IBD (Isolation By Distance): a program for analyses of isolation by distance.. J Hered.

[71] Akaike H, Second International Symposium on Information Theory, editors (1973). Information theory as an extension of the maximum likelihood principle.. Petrov BN and Csaki F.

[72] Burnham KP, Anderson DR (1998). Model selection and inference: a pratical information theoretic approach.

[73] Hurvich CM, Tsai CL (1989). Regression and time series model selection in small samples.. Biometrika.

[74] Legendre P (1993). Spatial autocorrelation: trouble or new paradigm?.. Ecology.

[75] Foll M, Gaggiotti O (2006). Identifying the environmental factors that determine the genetic structure of populations.. Genetics.

[76] Kuehn R, Hindenlang KE, Holzgang O, Senn J, Stoeckle B (2007). Genetic effect of transportation infrastructure on roe deer populations (*Capreolus capreolus*).. J Hered.

[77] Pérez-Espona S, Pérez-Barbería FJ, McLeod JE, Jiggins CD, Gordon IJ (2008). Landscape features affect gene flow of Scottish Highland red deer (*Cervus elaphus*).. Mol Ecol.

[78] Liu B, Jiang Z (2004). Dietary overlap between Przewalski's gazelle and Domestic sheep in the Qinghai Lake region and its implication for rangeland management.. J Wildl Manage.

[79] Li Z, Jiang Z, Li C (2008). Dietary overlap of Przewalski's gazelle, Tibetan gazelle and Tibetan sheep on the Qinghai-Tibet plateau.. J Wildl Manage.

[80] Li C, Jiang Z, Feng Z, Yang X, Yang J (2009). Effects of highway traffic on diurnal activity of the critically endangered Przewalski's gazelle.. Wildl Res.

[81] Hu J, Ping X, Cai J, Li Z, Li C (2010). Do local communities support the conservation of endangered Przewalski's gazelle?. Eur J Wildl Res.

