# Supplementary figures and images for: Effect of Anthropogenic Landscape Features on Population Genetic Differentiation of Przewalski's Gazelle: Main Role of Human Settlement
Source: PLoS One. 2011 May 20;6(5):e20144. doi: 10.1371/journal.pone.0020144 (PMC3098875; doi:10.1371/journal.pone.0020144)

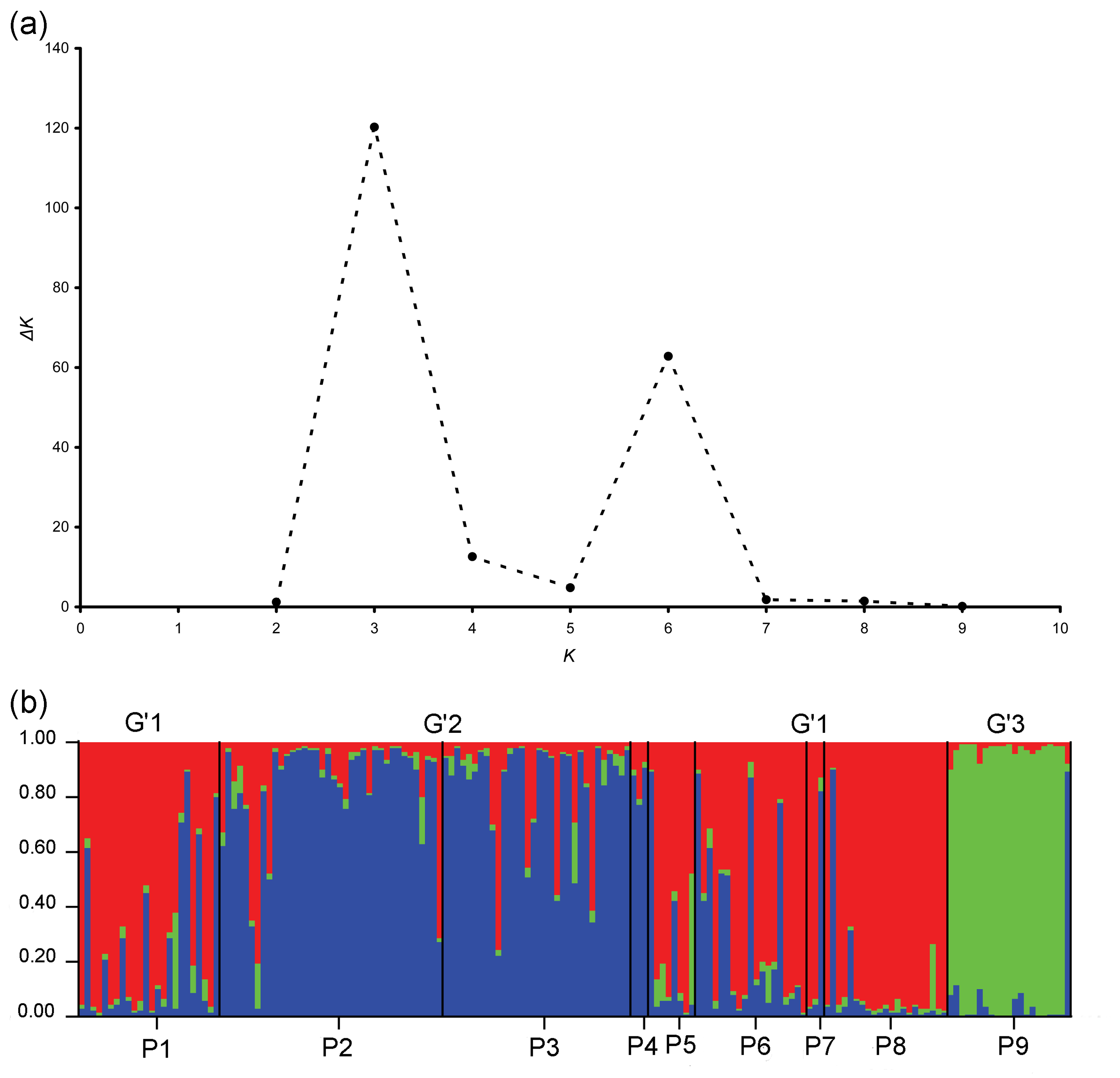

Supplement: Figure S1 — Output of Structure analysis according to ΔK values. (a) Plot of ΔK indicates that there are most likely three genetic groups. (b) Bar plot of three genetic groups. The sampling populations for individuals are shown as P1–P9, and the genetic groups assigned (G′1–G′3) are shown above: G′1 including P1, P5, P6, P7 and P8–red; G′2 including P2, P3 and P4–blue; G′3 including P9–green. (TIF) [file pone.0020144.s001.tif]

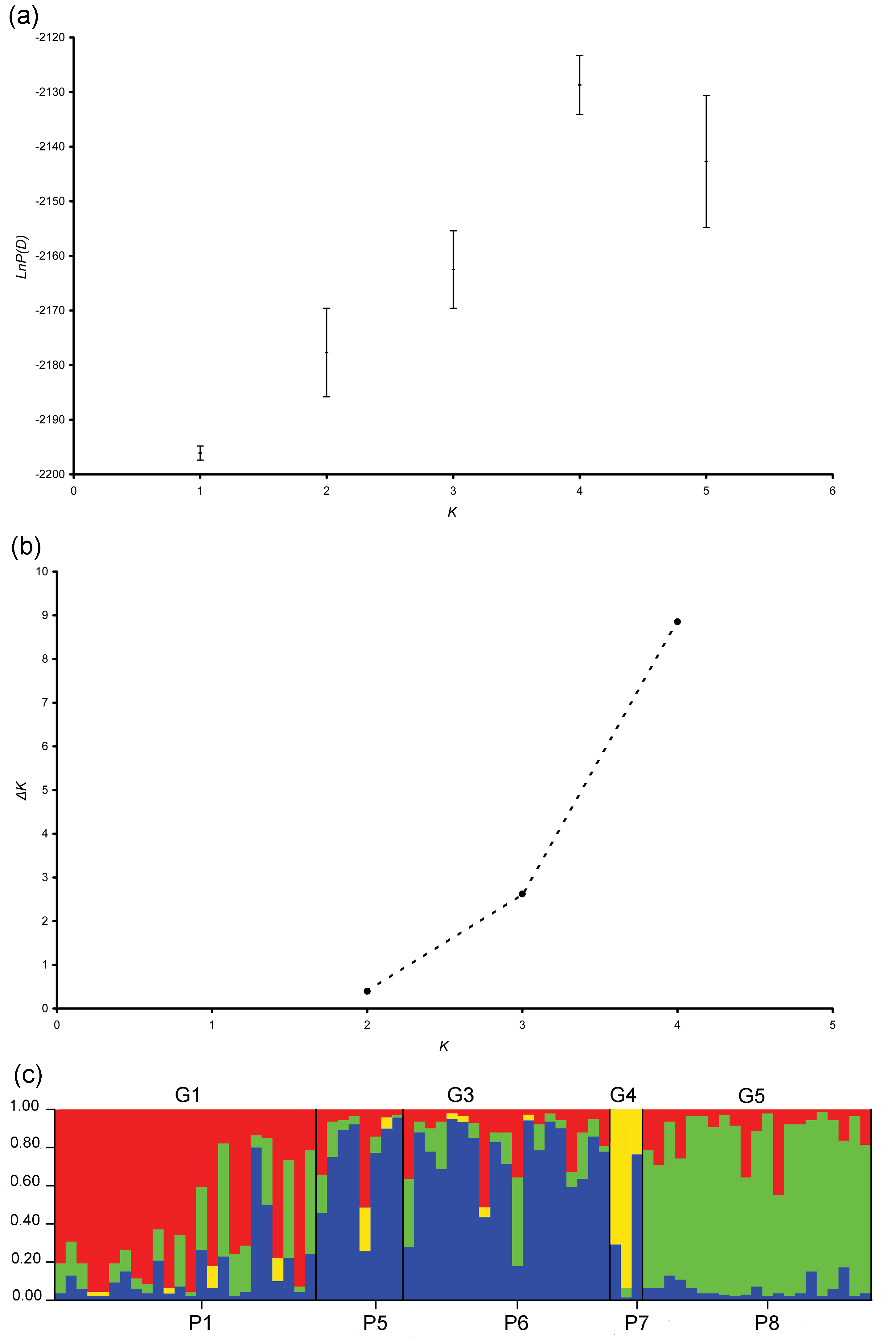

Supplement: Figure S2 — Output of Structure analysis using the data of G′1. (a) Average values of Ln P(D) show that the highest log likelihood occurs at K = 4 genetic groups. (b) Plot of ΔK indicates that there are most likely four genetic groups. (c) Bar plot of four genetic groups. The sampling populations for individuals are shown as P1, P5, P6, P7 and P8, and the genetic groups assigned are shown above: G1 including P1–red; G3 including P5 and P6–blue; G4 including P7–yellow; G5 including P8–green. (TIF) [file pone.0020144.s002.tif]
