# Supplementary material for: Effect of Anthropogenic Landscape Features on Population Genetic Differentiation of Przewalski's Gazelle: Main Role of Human Settlement
Source: PLoS One. 2011 May 20;6(5):e20144. doi: 10.1371/journal.pone.0020144 (PMC3098875; doi:10.1371/journal.pone.0020144)
Supplement: Table S1 — Matrix of categorical distance of railway (lower diagonal) between nine populations. (DOC) [file pone.0020144.s003.doc]

**Table S1. Matrix of categorical distance of railway (lower diagonal) between nine populations.**

| Population | P1 | P2 | P3 | P4 | P5 | P6 | P7 | P8 | P9 |
| --- | --- | --- | --- | --- | --- | --- | --- | --- | --- |
| P1 | — |  |  |  |  |  |  |  |  |
| P2 | 0 | — |  |  |  |  |  |  |  |
| P3 | 0 | 0 | — |  |  |  |  |  |  |
| P4 | 0 | 0 | 0 | — |  |  |  |  |  |
| P5 | 0 | 0 | 0 | 0 | — |  |  |  |  |
| P6 | 1 | 1 | 1 | 1 | 1 | — |  |  |  |
| P7 | 0 | 0 | 0 | 0 | 0 | 1 | — |  |  |
| P8 | 1 | 1 | 1 | 1 | 1 | 1 | 1 | — |  |
| P9 | 0 | 0 | 0 | 0 | 0 | 1 | 0 | 1 | — |

Categorical distances described the presence (1) or absence (0) of the landscape feature between two populations.
