# Supplementary material for: Effect of Anthropogenic Landscape Features on Population Genetic Differentiation of Przewalski's Gazelle: Main Role of Human Settlement
Source: PLoS One. 2011 May 20;6(5):e20144. doi: 10.1371/journal.pone.0020144 (PMC3098875; doi:10.1371/journal.pone.0020144)
Supplement: Table S2 — The sample size and genetic diversity within each population. (DOC) [file pone.0020144.s004.doc]

**Table S2.** The sample size and genetic diversity within each population.

| Population | Sample size | *H_S_* |
| --- | --- | --- |
| P1 | 24 | 0.574 |
| P2 | 38 | 0.473 |
| P3 | 32 | 0.462 |
| P4 | 3 | 0.468 |
| P5 | 8 | 0.533 |
| P6 | 19 | 0.532 |
| P7 | 3 | 0.641 |
| P8 | 21 | 0.553 |
| P9 | 21 | 0.564 |

*H_S_*, within-population genetic diversity, equal to the expected heterozygosity.
