# Supplementary material for: Effect of Anthropogenic Landscape Features on Population Genetic Differentiation of Przewalski's Gazelle: Main Role of Human Settlement
Source: PLoS One. 2011 May 20;6(5):e20144. doi: 10.1371/journal.pone.0020144 (PMC3098875; doi:10.1371/journal.pone.0020144)
Supplement: Table S3 — Evaluation of seven organizational models when using the genetic distance F′ST/(1−F′ST) in Mantel tests. (DOC) [file pone.0020144.s005.doc]

**Table S3.** Evaluation of seven organizational models when using the genetic distance *F'_ST_*/(1 − *F'_ST_*) in Mantel tests.

| Organizational model | Expectation | | *P* value | Support rate |
| --- | --- | --- | --- | --- |
| Model 1 isolation by human settlement | HG. R | S | **0.015** | 1.00 |
|  | HG. T | S | **0.014** |  |
|  | RG. H | NS | **0.102** |  |
|  | TG. H | NS | **0.093** |  |
| Model 2 isolation by human settlement and road | HG. R | S | **0.015** | 0.67 |
|  | HG. T | S | **0.014** |  |
|  | RG. H | S | 0.102 |  |
|  | RG. T | S | 0.053 |  |
|  | TG. H | NS | **0.093** |  |
|  | TG. R | NS | **0.069** |  |
| Model 3 isolation by human settlement and topography | HG. R | S | **0.015** | 0.67 |
|  | HG. T | S | **0.014** |  |
|  | TG. H | S | 0.093 |  |
|  | TG. R | S | 0.069 |  |
|  | RG. H | NS | **0.102** |  |
|  | RG. T | NS | **0.053** |  |
| Model 4 isolation by human settlement, road and topography | HG. R | S | **0.015** | 0.33 |
|  | HG. T | S | **0.014** |  |
|  | RG. H | S | 0.102 |  |
|  | RG. T | S | 0.053 |  |
|  | TG. H | S | 0.093 |  |
|  | TG. R | S | 0.069 |  |
| Model 5 isolation by road | RG. H | S | 0.102 | 0.25 |
|  | RG. T | S | 0.053 |  |
|  | HG. R | NS | 0.015 |  |
|  | TG. R | NS | **0.069** |  |
| Model 6 isolation by topography | TG. H | S | 0.093 | 0.25 |
|  | TG. R | S | 0.069 |  |
|  | HG. T | NS | 0.014 |  |
|  | RG. T | NS | **0.053** |  |
| Model 7 isolation by road and topography | RG. H | S | 0.102 | 0.00 |
|  | RG. T | S | 0.053 |  |
|  | TG. H | S | 0.093 |  |
|  | TG. R | S | 0.069 |  |
|  | HG. R | NS | 0.015 |  |
|  | HG. T | NS | 0.014 |  |

H = human settlement, R = road, T = topography, G = genetic distance. The period in the expectation abbreviations separate the covariate matrix from the two primary matrices. For example, HG. R indicates a partial Mantel test between the human settlement and genetic matrices, with the road matrix partialed out. Boldface indicates that the *P* value matches the expectations of the model. S = significant, NS = not significant, *P* < 0.05 indicates significant.
